# Comparison of core-genome MLST, coreSNP and PFGE methods for *Klebsiella pneumoniae* cluster analysis

**DOI:** 10.1099/mgen.0.000347

**Published:** 2020-03-09

**Authors:** Floriana Gona, Francesco Comandatore, Simone Battaglia, Aurora Piazza, Alberto Trovato, Giovanni Lorenzin, Paola Cichero, Anna Biancardi, Paola Nizzero, Matteo Moro, Daniela Maria Cirillo

**Affiliations:** ^1^​ Emerging Bacterial Pathogens Unit, Division of Immunology, Transplantation and Infectious Diseases, IRCCS San Raffaele Scientific Institute, Milan, Italy; ^2^​ Department of Biomedical and Clinical Sciences “L. Sacco”, University of Milan, Pediatric Clinical Research Center “Romeo and Enrica Invernizzi”, Milan, Italy; ^3^​ Institute of Microbiology and Virology, Department of Biomedical, Surgical and Dental Sciences, University of Milan, Milan, Italy; ^4^​ Laboratory of Microbiology and Virology, IRCCS San Raffaele Scientific Institute, Milan, Italy; ^5^​ Infection Control, IRCCS San Raffaele Scientific Institute, Milan, Italy

**Keywords:** *K. pneumoniae*, cgMLST, CG258, cluster

## Abstract

In this work we compared the most frequently used *Klebsiella pneumoniae* typing methods: PFGE, cgMLST and coreSNP. We evaluated the discriminatory power of the three methods to confirm or exclude nosocomial transmission on *K. pneumoniae* strains isolated from January to December 2017, in the framework of the routine surveillance for multidrug-resistant organisms at the San Raffaele Hospital, in Milan. We compared the results of the different methods to the results of epidemiological investigation. Our results showed that cgMLST and coreSNP are more discriminant than PFGE, and that both approaches are suitable for transmission analyses. cgMLST appeared to be inferior to coreSNP in the *K. pneumoniae* CG258 phylogenetic reconstruction. Indeed, we found that the phylogenetic reconstruction based on cgMLST genes wrongly clustered ST258 clade1 and clade2 strains, conversely properly assigned by coreSNP approach. In conclusion, this study provides evidences supporting the reliability of both cgMLST and coreSNP for hospital surveillance programs and highlights the limits of cgMLST scheme genes for phylogenetic reconstructions.

## Data Summary

1.Sequence read files for all 80 isolates have been deposited in SRA, accessible through NCBI BioSample accession numbers and whole-genome shotgun projects have been deposited in Genbank (BioProject PRJNA564099 for K. pneumoniae)

2.A full list of SRA run accession numbers (Illumina reads) for these samples are available in Table S2 (available in the online version of this article).

**Impact Statement**

*
K. pneumoniae* is one of the most common causes of healthcare-associated infections. The global spread of carbapenemase-producing *K. pneumoniae* high-risk clones is a public health concern. In the last decade, most hospital outbreaks of carbapenem-resistant *K. pneumoniae* have been attributed to *K. pneumoniae* carbapenemase (KPC)-producing isolates belonging to clonal group (CG) 258. Like many research and public health laboratories, we frequently perform large-scale bacterial comparative genomics studies using Illumina sequencing, which assays gene content and provides the high-confidence variant calls needed for phylogenomics and transmission studies. We compared the most frequently used *K. pneumoniae* typing methods: PFGE, cgMLST and coreSNP. We compared the results of the different methods to the results of epidemiological investigation. Our results showed that cgMLST and coreSNP are more discriminant than PFGE, and that both approaches are suitable for transmission analyses. cgMLST appeared to be inferior to coreSNP in the *K. pneumoniae* CG258 phylogenetic reconstruction.

## Introduction

*Klebsiella pneumoniae* carbapenemase-producing *K. pneumoniae* (KPC-Kp) is a major cause of healthcare-associated infections (HAIs). Reported estimates show that the mortality rate among patients with KPC-Kp bloodstream infections ranges from 40 to 70 %, while for patients with KPC-Kp pneumonia ranges from 20 to 40 % [1, 2]. In many countries, including Italy, KPC-Kp has reached endemic proportions [3–5]. The majority of the KPC-Kp isolated worldwide belong to the clonal group CG258, a well-demarcated group of strains defined on the basis of cgMLST profiles [3]. CG258 strains belong to several sequence type (STs) (defined by the multi-locus sequence type scheme, https://pubmlst.org/software/database/bigsdb/), including the high-risk ST258, ST11, ST512 and ST340 [6]. The clone ST258 predominates largely in North America, Latin America and Europe, while the clone ST11 is much more prevalent in Asia and Latin America [7]. The clone ST512 is frequently isolated in Italy, Colombia, and Israel while the clone ST340 is common in Brazil and Greece [8]. An epidemic dissemination of KPC-Kp has been reported in Italy since 2010 mostly related to the spread of the ST258 clone [9]. The emergence of new clones contributed to increase the genetic diversity in all countries, as described in a recent European study [10]. The strategies for the detection and surveillance of KPC-Kp-circulating clones have received significant attention in recent years [11, 12].

Several molecular methods have been proposed for *K. pneumoniae* typing in outbreak and cluster investigations [13, 14]. Criteria for the evaluation of typing methods' performance include reproducibility, discriminatory power and epidemiological concordance [15].

PFGE is still the gold standard technique to investigate the relatedness among isolates and to support epidemiological investigations. However, due to the high clonality of *K. pneumoniae* clinical isolates (most of which belong to CG258), this method may not provide sufficient resolution power to distinguish clusters, thus reducing the ability to discern transmission dynamics [16]. Recently, methods based on whole-genome sequencing (WGS) have been used to trace phylogenetic relationships and to identify *K. pneumoniae* clones [17–19].

The most common way to compare genomes is to evaluate the differences in SNPs [14]. An alternative approach is the core-genome MLST (cgMLST): an improvement of the MLST concept to the genome level [20–22]. cgMLST schemes contain hundreds to thousands of core genes showing a discriminatory power higher than MLST schemes, which include only few genes (e.g. seven for the *K. pneumoniae* scheme). Two different cgMLST schemes are available for *K. pneumoniae *: BIGSdb [21] and SeqSphere+ (http://www.cgmlst.org/ncs). In the literature, the latter is the most frequently used, probably because a standalone and user-friendly software is available [23–25].

Previous studies on other bacterial species have been performed to evaluate the concordance between cgMLST and coreSNP methods [21, 26, 27]. Despite the clinical relevance of *K. pneumoniae,* only few evidences [23, 25, 26] and no specific studies on this topic are present in the literature.

The aim of this study is to compare the three most frequently used *K. pneumoniae* typing methods: PFGE, cgMLST (SeqSphere+) and coreSNP. We also evaluated the concordance of results on the transmission events of carbapenem-resistant *K. pneumoniae* among patients admitted at the San Raffaele hospital (OSR), in Milan, during 2017. Furthermore, we compared the phylogenetic signal of cgMLST and coreSNP on a large genomic dataset including the genomes of the *K. pneumoniae* strains collected during the OSR surveillance program and ~400 genomes retrieved from public database [28].

## Methods

### Isolate collection

The strains included in this retrospective study were collected from January to December 2017 in the framework of the routine surveillance for multidrug-resistant organisms in place at the San Raffaele Hospital in Milan (OSR). The strains originating from duplicates from the same patient were excluded.

Cultures for isolation of carbapenem-resistant (CR-KP) were performed on MacConkey agar plates containing a 10 µg disk of carbapenem. After 24–48 h of incubation at 37 °C, the colonies growing close to the disk were collected and identified by MALDI-TOF mass spectrometry (Vitek MS bioMérieux, Florence, Italy).

An antimicrobial sensitivity testing was performed by automated microdilution using the Vitek-2 AST-GN202 card and imipenem and meropenem MICs were verified with the E-test. Resistance mechanisms were confirmed by phenotypic assays: the 'modified Hodge test' was used to detect carbapenemase activity, synergy between phenyl-boronic acid and carbapenems in combined disk tests were used to detect KPC-Kp, and synergy between EDTA and carbapenems in combined disk were used to detect metallo-β-lactamases. All *K. pneumoniae* strains positive to carbapenemase phenotypic test were processed for WGS and PFGE.

### PFGE

Briefly, genomic DNA was digested with *XbaI* enzyme and run into a CHEF-DRIII system, as previously described [29, 30]. PFGE profiles and cluster analyses were identified by using the software InfoQuest FP version 5.1 (Bio-Rad, Hercules, CA, USA) and confirmed by the epidemiological investigation. A cluster was defined as two or more related KPC-Kp cases presenting the same clone, according to the molecular-typing results, and a link confirmed if those patients had shared the same ward for at least 1 day in intensive care units; for at least 2 calendar days in any other ward, limiting the investigation to the current hospitalization, irrespective to the date of isolation of the KPC-Kp and of its length.

### WGS

For DNA extraction, bacterial cultures were purified by two successive single colony selections after streaking on blood agar medium incubated overnight at 37 °C (Becton Dickinson, Franklin Lakes, NJ, USA). Bacterial DNA was extracted from a liquid suspension of the purified cultures using the Maxwell 16 Cell DNA Purification Kit SEV in combination with a Maxwell 16 Instrument (Promega, USA). All strains were sequenced by Illumina NextSeq500 platform, (Illumina, San Diego, CA, USA), with a paired-end run of (2×150 bp), after Nextera XT paired-end library preparation following the manufacturer’s instructions [31].

Sequencing reads were *de novo* assembled using SPAdes (version 3.13) [32]. WGS data were used for genotypic characterization and virulence-gene detection by blast search using gene datasets available at the Bacterial Isolate Genome Sequence Database (BIGSdb) [21]. We will refer to this genome dataset as ‘OSR dataset’.

### Genome-dataset reconstruction and sequence-type profile determination

We reconstructed a *K. pneumoniae* background genomic dataset as follows. We retrieved all the 924 *K *. *pneumoniae* genome assemblies present in the PATRIC database on 29 October 2018 for which the publication code was available (in accordance with Fort Lauderdale and Toronto agreements). Then we selected a subset of these retrieved assemblies on the basis of their genetic distances from the OSR assemblies. In more detail, we computed the genetic distance between each OSR genome assembly and each retrieved genome assembly using Mash software [33]. For each OSR genome assembly, we selected the 50 less distant assemblies retrieved from PATRIC. Lastly, selected PATRIC assemblies and OSR assemblies were merged in a dataset called ‘Global dataset’.

In order to exclude low-quality selected PATRIC assemblies (i.e. >300 contigs or genome size not compatible with complete assemblies of *K. pneumoniae *) we assessed the number of contigs and the total genome length using quast software [34] (Table S1). The maximum contigs number was 240, and the ranges of total length between complete assemblies and scaffolds were comparable (total length: 5 118 878–6 107 937 and 4 988 911–5,835,446, for complete and scaffold assemblies, respectively). Thus, no genome assemblies were excluded from the analysis.

The MLST profiles of all the strains included in the study (i.e. those sequenced in this work and those retrieved from the PATRIC database) were *in silico* determined using an *in-house* Perl script (https://skynet.unimi.it/index.php/tools/purple-tool/). The MLST gene sequences and profiles used for the analyses were retrieved from the BIGSdb database.

### Core-genome MLST

For OSR dataset, core-genome MLST (cgMLST) analysis was performed using SeqSphere+software (6.0.0 version Ridom, GmbH, Münster, Germany) according to the ‘*K. pneumoniae sensu lato* cgMLST’ version 1.0 scheme (https://www.cgmlst.org/ncs/schema/2187931/). This comprises a total of 2358 genes (about 40 % of the NTUH-K2044 reference genome) [31]. SeqSphere+tool was used to map the reads against the reference genome using BWA v 0.6.2 software (parameters setting: minimum coverage of five and Phred value >30) and to determine the cgMLST gene alleles. The combination of all these alleles in each strain formed an allelic profile that was used to generate minimum spanning tree (MST) using SeqSphere+with the ‘pairwise ignore missing values’ parameter. A threshold of ≤4 allelic differences was used to define the clusters [31, 35, 36].

Assembled reads from 486 genomes present in PATRIC were imported to Seqsphere+ and the target scan procedure was performed by using the built-in blast v 2.2.12 for cgMLST analysis.

The cgMLST gene concatenate of the OSR dataset and Global dataset were obtained as follows: (i) the cgMLST genes, for which the variants were determined in all the strains, were selected; (ii) for each selected gene, the sequences relative to the named variants were retrieved from the SeqSphere+cgMLST gene dataset and aligned using muscle [37]; (iii) the obtained gene alignments were concatenated using an *in-house* Perl script (https://drive.google.com/open?id=1OlSmcQmcm4-5hfSCu1bov3M8AXS96Xbt).

### CoreSNPs calling and clustering

The coreSNP calling analysis was performed for both OSR and Global datasets. All the assemblies included in the dataset were aligned to the *K. pneumoniae* reference genome NTUH-K2044 using progressiveMauve [38] and the coreSNP calling was performed as described by Gaiarsa and colleagues [39]. CoreSNPs were definied as gap-free variable positions of the alignment flanked, on the right and on the left, by at least five conserved positions. CoreSNPs localized inside repeated regions (identified using MUMmer, [40]) or phages (identified using phiSpy, [41]) on the reference assembly were masked. This approach has been previously used in surveillance studies [42] and outbreak reconstructions[43]. OSR strains were then clustered in groups with cutoff <21 SNPs, as previously described [43]. Then the coreSNP-based MST was computed using the R library Ape [44].

### Phylogenetic analyses and comparison

For both the ‘OSR’ and ‘Global datasets’, cgMLST concatenates and coreSNP alignments were subjected to the best model selection using ModelTest-NG following the Bayesian information criterion (BIC) [45]. For OSR and Global cgMLST alignments the best model resulted GTR, while for OSR and Global coreSNP alignments the best model resulted TVM. For each alignment, maximum likelihood (ML) phylogenetic analyses were perfomed using RaxML8 software [46] with 100 pseudo-bootstraps and the relative selected model. Furthermore, distance matrix of cgMLST concatenate and of coreSNP alignment were computed using the R library Ape and compared using the Mantel test.

## Results

### Bacterial strain description: KPC variants and sequence-type distribution

A total of 80 carbapenem-resistant *K. pneumoniae* isolates were collected during the study period and included in the present work. Most of the strains (55/80, 69 %), were isolated from diagnostic specimens. Among them, 15 derived from urine samples, 9 from respiratory samples, 13 were abdominal wound samples and 17 from blood samples. The remaining 25 isolates (31 %) were isolated from perirectal swabs collected for surveillance purposes. The overall results from WGS analyses and the genotypic characterization of the 80 *K *. *pneumoniae* strains are reported in Table 1. All isolates carried *bla*_KPC-3_ (*n*=67) or *bla*_KPC-2_ (*n*=13), no other class A enzyme genes (*bla*_SME_, *bla*_IMI_) or metallo beta-lactamase genes (including *bla*_NDM_ or *bla*_VIM_) were detected.

**Table 1.** Sequence-type distribution, KPC variants and virulence factors

ST	n° strains	*wzi*	*k-type*	*Virulence factors*								KPC
**37**	1	96	*K38*				–	–	–	*mrkABCD*	–	**KPC-3**
**11** (**CG258**)	1	75	–	*fyuA*	*irp1*	*irp2*	–	–	–	*mrkABCD*	*ybtAEPQSTUX*	**KPC-2**
**101**	3	137	*K17*	*fyuA*	*irp1*	*irp2*	–	–	*kFuABC*	*mrkABCD*	*ybtAEPQSTUX*	**KPC-2**
**101**	2	137	*K17*	*fyuA*	*irp1*	*irp2*	–	–	*kFuABC*	*mrkABCD*	*ybtAEPQSTUX*	**KPC-3**
**149**	1	62	*K62*	*fyuA*	*irp1*	*irp2*	–	–	–	*mrkABCD*	*ybtAEPQSTUX*	**KPC-3**
**258** (**CG258**)	2	29 (*cps-1*)	–		*irp1*	*irp2*	–	–	–	*mrkABCD*	*ybtAEPQSTUX*	**KPC-2**
**258** (**CG258**)	3	154 (*cps-2*)	–	–	–	–	–	–	–	*mrkABCD*	–	**KPC-3**
**512** (**CG258**)	31	154 (*cps-2*)	–	–	–	–	–	–	–	*mrkABCD*	–	**KPC-3**
**307**	27	173	–	*fyuA*	*irp1*	–	–	–	–	*mrkABCD*	*ybtAEPQSTUX*	**KPC-3**
**307**	5	173	–	*fyuA*	*irp1*	–	–	–	–	*mrkABCD*	*ybtAEPQSTUX*	**KPC-2**
**395**	2	2	*K2*	*fyuA*	*irp1*	*irp2*	*iucABCD*	*iutA*		*mrkABCD*	*ybtAEPQSTUX*	**KPC-3**
**15**	1	89	–	*fyuA*	*irp1*	*irp2*	–	–	*kFuABC*	*mrkABCD*	*ybtAEPQSTUX*	**KPC-3**
**423**	1	8	*K8*	*fyuA*	*irp1*	*irp2*	–	–	–	*mrkABCD*	*ybtAEPQSTUX*	**KPC-3**

The most represented MLST lineage is the clonal group CG258 (*n*=37), followed by ST307 (*n*=32) and ST101 (*n*=5). Among the CG258 strains, all the ST512 (*n*=31) harboured the KPC-3 variant. Three of the the five ST258 strains, presented the *bla*_KPC-3_ genes and two the *bla*_KPC-2_ genes. The ST11 isolate carried the KPC-2 variant. Finally, among ST307 strains both the *bla*_KPC-2_ and the *bla*_KPC-3_ genes were identified in 5 and 27 isolates, respectively (see Table 1).

All investigated isolates harboured *mrkABCDF*, *iucABCDiA* and *yersiniabactin* markers (irp and ybt). The regulators of the mucoid phenotype (an indicator of hypervirulence, rmpA and rmpA2) and two capsular serotypes, K1 and K2, were absent in our isolates. The *cps*-1 and *cps*-2 capsular gene clusters were restricted to CG258 strains. The *cps*-2 capsular type was found to be associated with isolates belonging to ST258 and ST512 (*n*=34), while *cps*-1 was preferentially associated with ST258 strains only (see Table 1). ST101 isolates carried the *wzi137* variant associated with the K17 serotype. ST307 strains carried *wzi173*, ST11 carried *wzi75* and ST15 carried *wzi89,* not associated with specific K-serotypes (Table 1).

### Comparison of the discriminatory power of PFGE, cgMLST and coreSNP

According to the interpreting criteria described by Tenover *et al*. [30], the 80 clinical isolates of *K. pneumoniae* isolated from the OSR were grouped into four clonal patterns, named A, B, C and D. The four PFGE clones corresponded to the MLST STs: clone A corresponds to ST512, clone B to ST307, clone C to ST258 and clone D to ST101.

**Fig. 1.**
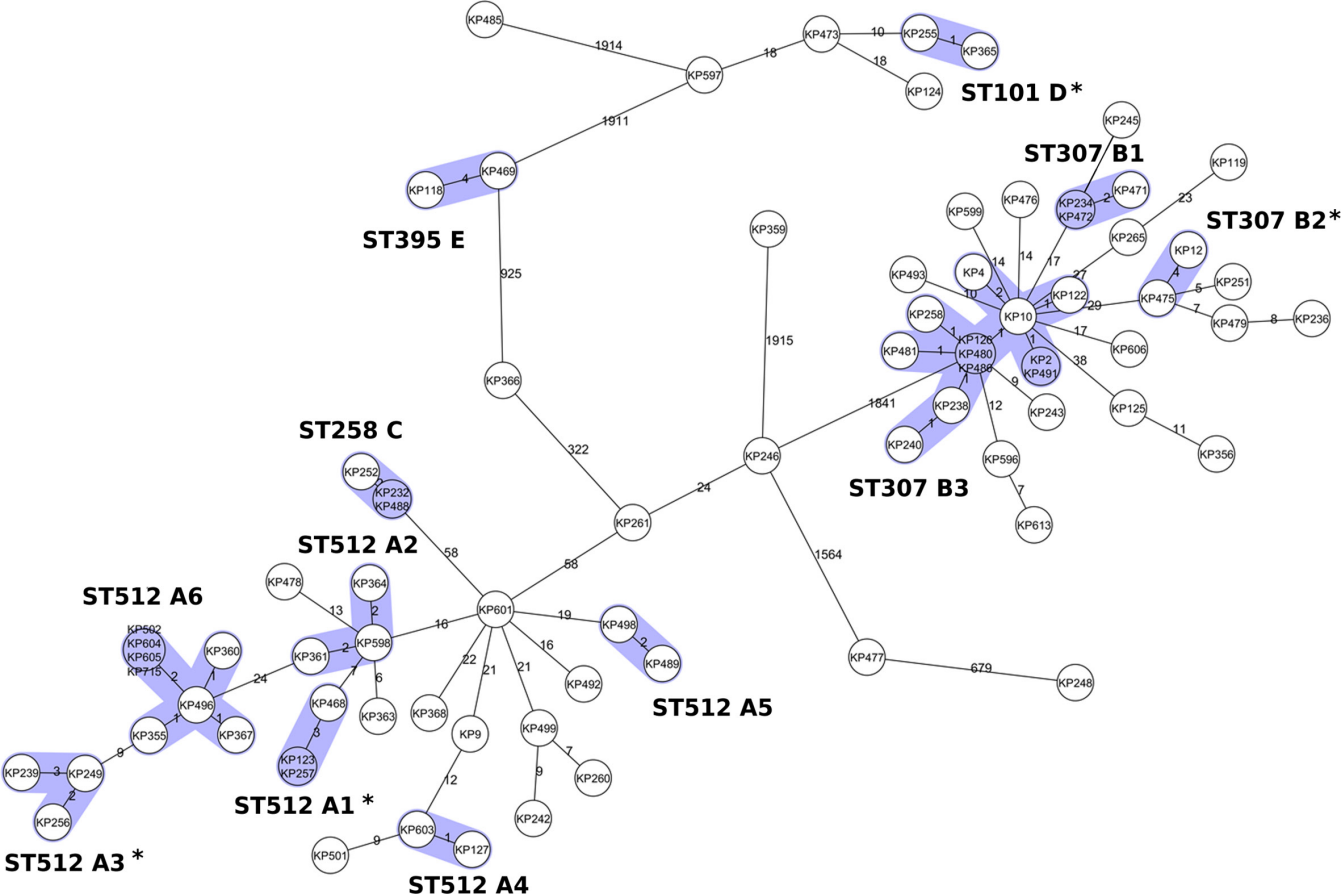
Clonal relationship of 80 *K *. *pneumoniae* isolates in a MST based on core-genome multilocus. Each circle represents a single genotype, i.e. an allelic profile based on up to 2358 target genes present in the isolates with the “pairwise ignoring missing values” option turned on in the SeqSphere^+^ software during comparison. The number on connecting lines represents the number of alleles that differ between the connected genotypes. The clusters identified on the cgMLST MST and absent in the MST computed on coreSNP MST (Fig. 2) are marked with asterisks.

The cgMLST cluster analysis grouped 44 out of the 80 isolates into 12 clonal clusters. The cgMLST grouped strains of the PFGE clonal pattern A into six different clusters (A1 to A6) and those of the clonal pattern B into three clusters (B1 to B3). The cgMLST clusters C and D correspond to the PFGE clonal patterns C and D. cgMLST allowed to identify a further cluster named E including two strains of ST395 (Fig. 1). cgMLST cluster A6 (eight ST512 strains) and B3 (12 ST307 strains) were dominant; the other ten clusters included only two or three isolates each (Fig. 1).

**Fig. 2.**
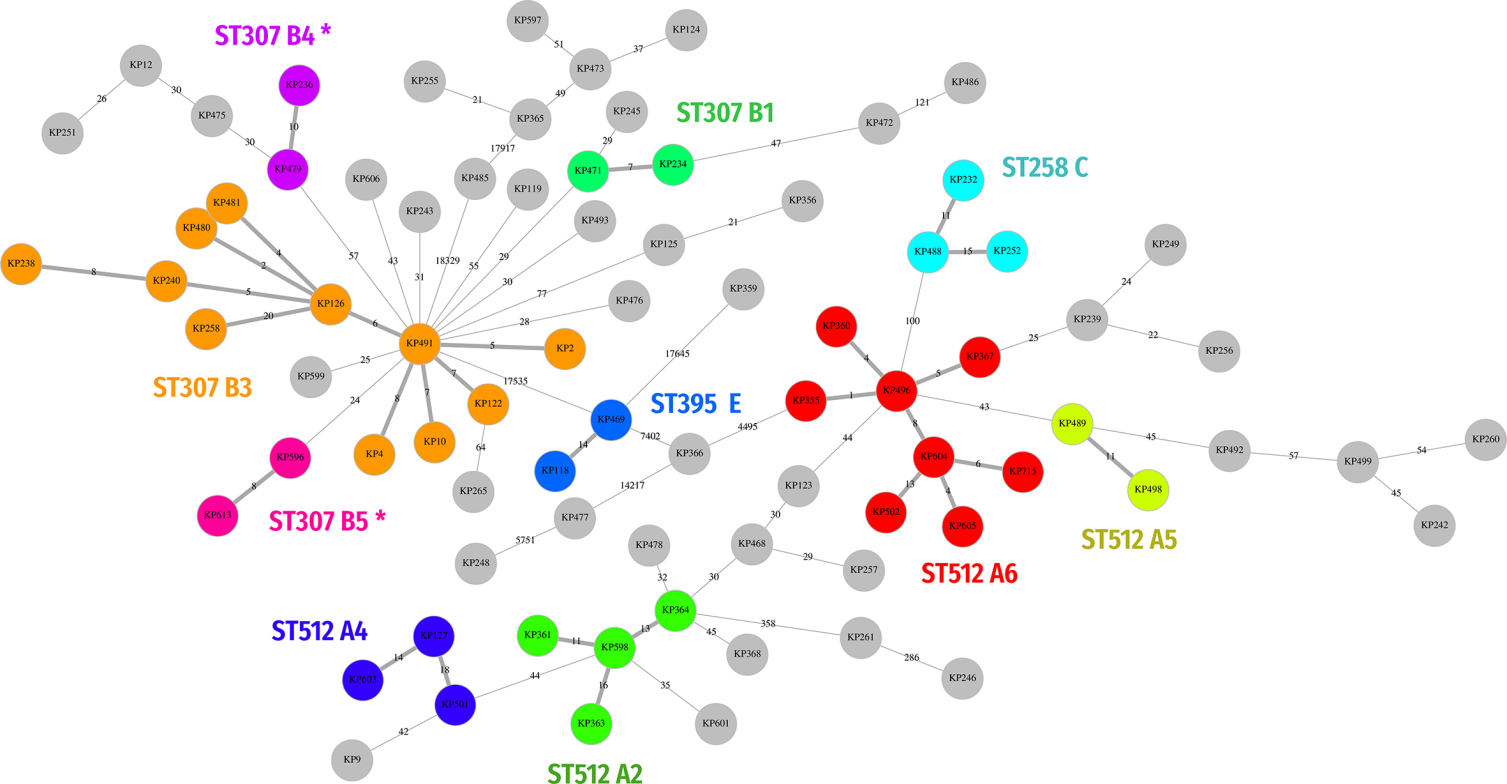
Clonal relationship of 80 *K *. *pneumoniae* isolates in a MST computed on coreSNP distances. Clusters of strains distant <21 SNPs have been identified, coloured and labelled on the graph. The clusters identified on the coreSNP MST and absent in the MST computed on cgMLST allele distances (Fig. 1) are marked with asterisks.

The coreSNP cluster analysis (with cutoff <21 SNPs) grouped 39 out of the 80 isolates into ten clonal clusters. Among these, eight were coherent with the cgMLST clusters: A2, A4, A5, A6, B1, B3, C and E. The remaining two clusters (B4 and B5) were detected only with coreSNP approach (Fig. 2). On the other hand, cgMLST identified four clusters not identified by coreSNP (A1, A3, B2 and D).

Epidemiological links among patients were investigated by the Infection Control Committee of the OSR for each cluster identified by PFGE, cgMLST or coreSNP. As shown in Table 2, *n*=18 epidemiological links were confirmed by the investigation, involving a total of 25 patients in six clusters. PFGE analysis failed to detect 10 out of 18 links, cgMLST two and coreSNP three.

**Table 2.** Description of the epidemiological links confirmed

Epidemiological links	Ward	Clusters	PFGE	cgMLST	coreSNP
KP360 - KP367	Neurosurgical Intensive Care Unit	**A6**		**+**	**+**
KP360 - KP496	Neurosurgical Intensive Care Unit	**A6**		**+**	**+**
KP367 - KP496	Neurosurgical Intensive Care Unit	**A6**	**+**	**+**	**+**
KP502 - KP605	Cardiosurgical Intensive Care Unit	**A6**		**+**	**+**
KP604 - KP715	Intensive Care Unit	**A6**		**+**	**+**

KP361 - KP363	Intensive Care Unit	**A2**	**+**	>4 alleles	**+**
KP363 - KP364	Intensive Care Unit	**A2**	**+**	>4 alleles	**+**
KP361 - KP364	Intensive Care Unit	**A2**	**+**	**+**	**+**

KP239 - KP249	Medicine	**A3**	**+**	**+**	>21 SNPs
KP249 - KP256	Medicine	**A3**	**+**	**+**	>21 SNPs

KP232 - KP252	Gastroenterological surgery	**C**		**+**	**+**
KP232 - KP488	Gastroenterological surgery	**C**		**+**	**+**
KP252 - KP488	Gastroenterological surgery	**C**		**+**	**+**

KP2 - KP4	Cardiosurgical Intensive Care Unit	**B**	**+**	**+**	**+**
KP258 - KP481	Cardiosurgical Intensive Care Unit	**B**	**+**	**+**	**+**
KP126 - KP480	Medicine	**B**		**+**	**+**
KP126 - KP491	Medicine	**B**		**+**	**+**

KP255 - KP365	Rehabilitation	**D**		**+**	>21 SNPs

### Comparison of phylogenetic reconstructions cgMLST and coreSNPs

CoreSNP alignment and cgMLST gene concatenates were obtained both for OSR and Global datasets, the first one including the 80 OSR genomes only and the second one including these 80 genomes and the other 406 selected from the PATRIC database (see Methods). The cgMLST concatenate lengths for the OSR and Global datasets were 902 289 bp and 440 658 bp, respectively. CoreSNP calling produced an alignment of 54 407 SNPs for OSR dataset and 85 676 SNPs for the Global one. This difference could be due to the different number of strains (80 for OSR and 486 for Global dataset) and thus to the different genetic variability inside the dataset. On the other hand, cgMLST concatenate of OSR is sized about twofold more than the Global dataset: we can explain it considering that cgMLST shared gene number (see Methods) decreases with the number of genomes (Fig. S1, Fig. S2). Indeed, for each genome we can expect that SeqSphere+ does not determine the allele variant of some genes (from here ‘undetermined genes’). The 90 % of the 486 strains of the Global dataset have less than 13 undetermined genes. The contig number and contig total length of the strains with <=13 undetermined genes and those with >13, resulted not significantly different (Wilcoxon test, contig num *P*-value=0.1 and contig total length *P*-value=0.5). This result suggests that the reduction in shared gene number is not due to the quality of the genome assemblies. Considering that a cgMLST variant can be called by SeqSphere+tool only if it is present in the cgMLST scheme, we can hypothesize that part of the ‘undetermined genes’ could be a consequence of the absence of some variants in the cgMLST SeqSphere+scheme.

The distance matrices computed on the cgMLST concatenate and coreSNP alignment are significantly correlated (Mantel test, *P*-value<0.001; Spearman test *R*=0.87, *P*-value<2.2 e-16, Fig. S3).

**Fig. 3.**
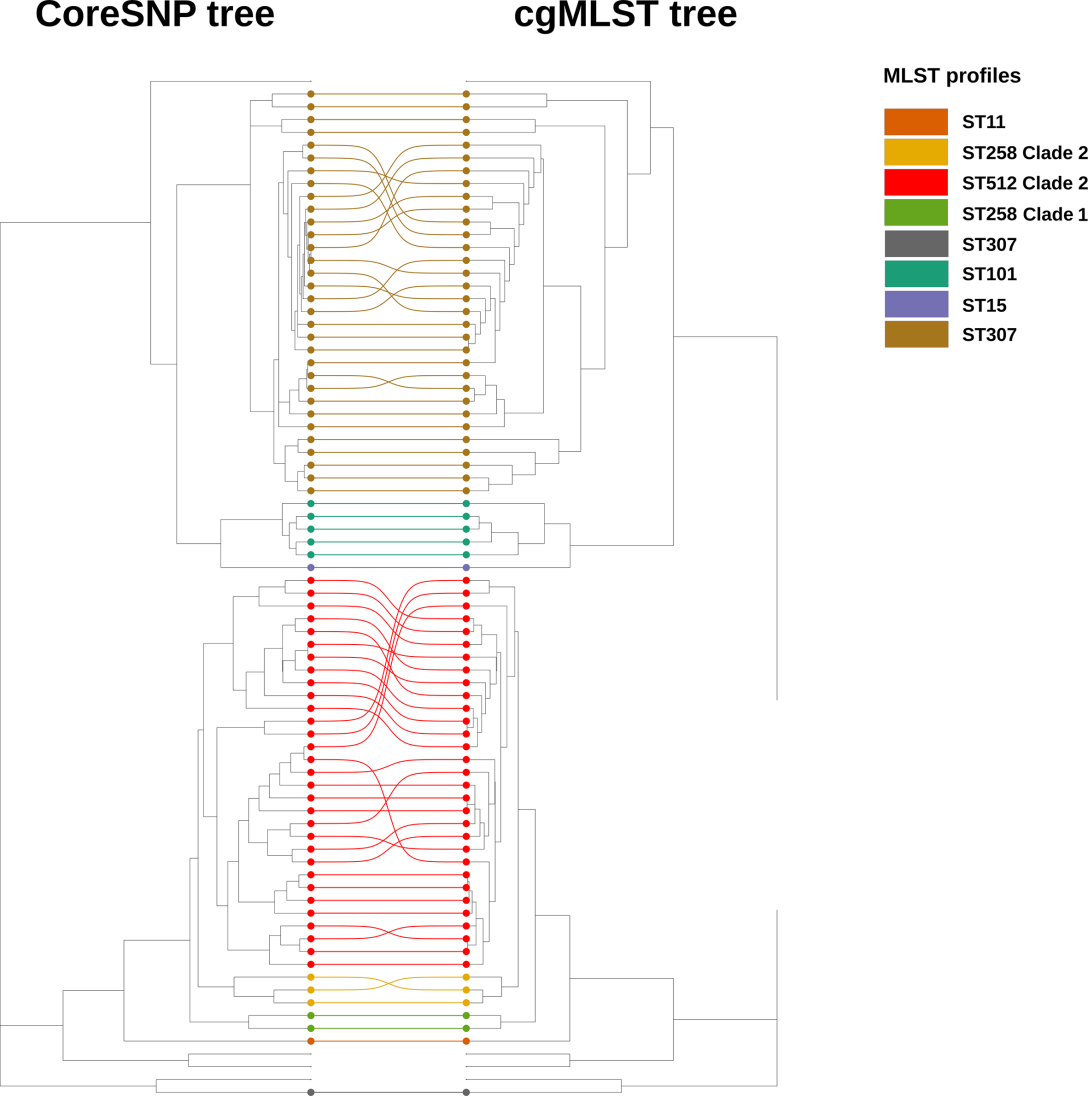
Comparison of the ML phylogenetic trees obtained with coreSNP and cgMLST on the OSR dataset (80 strains isolated during OSR routine surveillance program). On the left, the tree obtained with coreSNP and on the right the tree with SeqSphere+cgMLST. The strains belonging to highly represented MLST profiles (>=10 strains) are connected by coloured lines. Among these strains, those included in the routine surveillance program are highlighted on the trees with coloured dots.

As shown in Fig. 3, OSR cgMLST and coreSNP trees were highly congruent, indeed all the ST clades were consistently placed on the trees.

**Fig. 4.**
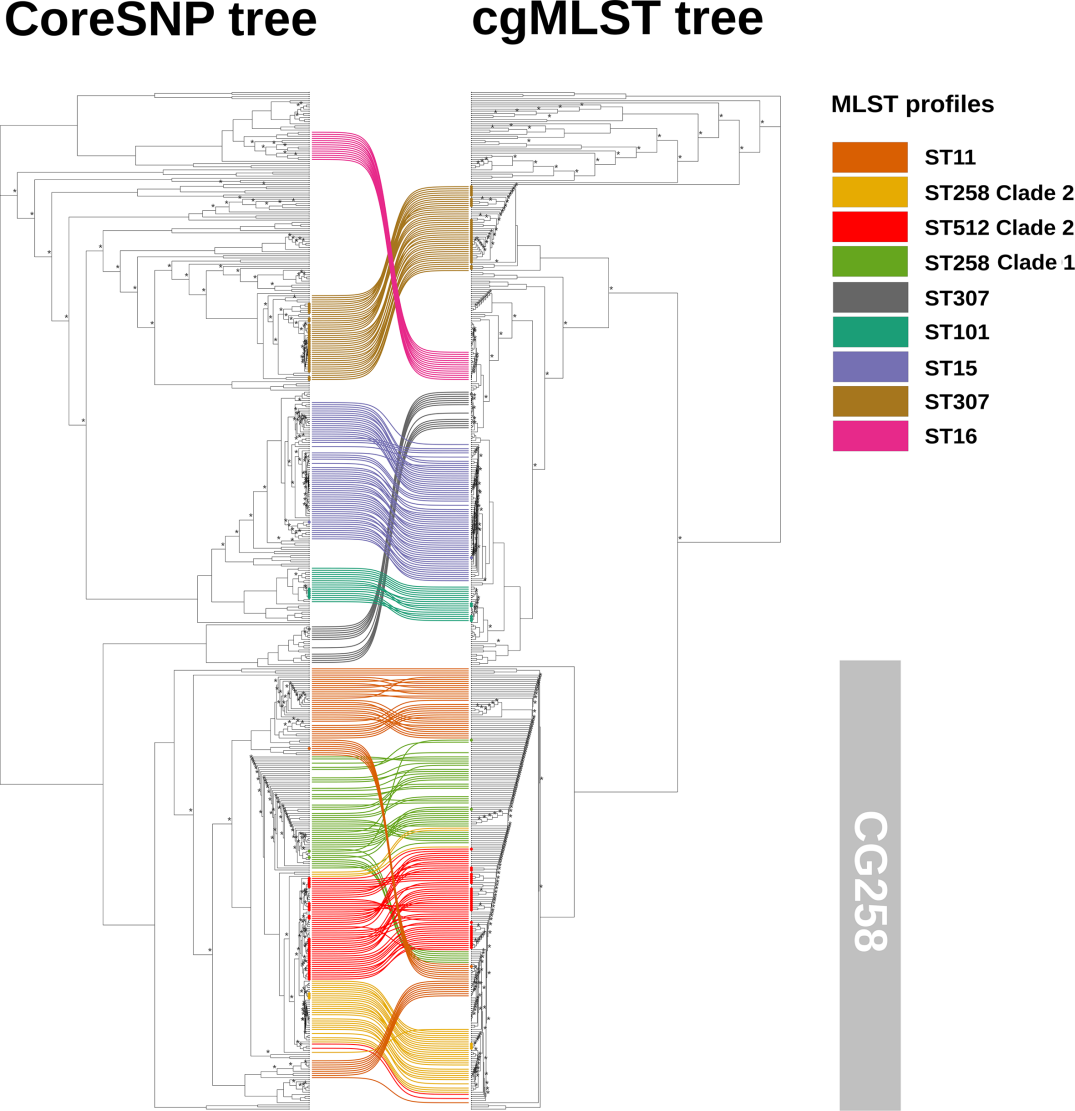
Comparison of the ML phylogenetic trees obtained with coreSNP and cgMLST on the Global dataset (486 *K. pneumoniae* strains: 80 isolated during OSR routine surveillance program and 406 from database). Asterisks are reported on nodes with bootstrap supports below 75. On the left, the tree obtained with coreSNP and on the right the tree with SeqSphere+cgMLST. The strains belonging to highly represented MLST profiles (>=10 strains) are connected by coloured lines. Among these strains, those included in the routine surveillance program are highlighted on the trees with coloured dots.

On the other hand, the two trees for the Global dataset were mainly coherent with exceptions within the CG258 (Fig. 4). The coreSNP tree correctly clustered the ST258_Clade 2 strains separating them from the ST258_Clade 1. Conversely, the cgMLST clustered the ST512 strains with ST258 _Clade 1 strains on the tree (Fig. 4). Finally, the ST11 strains were correctly placed as basal to the ST258 lineage (clade1 and clade2) by coreSNP tree, while the cgMLST tree places some ST11 strains as part of a separated clade including ST258_ Clade 2 and ST11 strains (Fig. 4).

Whole-genome shotgun projects have been deposited in Genbank (BioProject PRJNA564099 for *K. pneumoniae *) and the accession numbers can be found in Table S2.

## Discussion

WGS allows the entire sequence of a bacterial genome to be obtained with an affordable cost and a short turnaround time. This drastically increases the amount of information available to compare bacterial strains improving bacterial typing discriminatory power. The most frequently used WGS-based bacterial typing methods are based on SNP detection and cgMLST. In recent years, despite the coherence and reliability of the two methods for epidemiological purposes being investigated for several bacterial species, little information is available for *K. pneumoniae *, one of the most important nosocomial pathogens.

cgMLST has been successfully used to support infection-control measures [47–49] at hospital level and to perform surveillance of specific pathogens at global level [50]. Indeed, like MLST, it allows a large number of bacterial genomes to be analysed and it provides a standard strain nomenclature easily shareable in an international context [51]. The cgMLST schemes usually contain from hundreds to thousands of genes, which represent only a part of the entire genome. The SNP analysis, exploiting the entire genome positions (including also intergenic regions), allows very closely related strains to be discriminated, and consequently detailed epidemiological investigations to be performed [21].

Currently, the most important limit to the application of WGS-based methods in the hospital epidemiological surveillance is the absence of established guidelines for the identification of bacterial relatedness, guidelines similar to those available for pre-WGS typing methods, such as PFGE [48]. In this study, we used PFGE and WGS-based typing methods (including cgMLST and coreSNPs) to perform cluster analysis and to evaluate epidemiological links on the 80 KP-KPC strains in the framework of the routine surveillance for multidrug-resistant organisms at the San Raffaele Hospital, in Milan, during 2017.

We showed that both cgMLST and coreSNP give comparable results in the high majority of cases. Indeed, the strains of the clusters found by cgMLST only (A1, A3, B2 and D; see Fig. 1) are also close on the coreSNP MST, but they were not assigned to any cluster due to their distances slightly exceeded the <21 SNP threshold (Fig. 2). Similarly, the few strains identified by coreSNP only (B4 and B5; Fig. 2) show an allele distances (range five to eight alleles) just above the threshold (≤four alleles). These results clearly showed how the threshold choice is a key point for WGS-based epidemiological investigation and may be modified according to the specific epidemiological context. By comparing PFGE, cgMLST and coreSNP with the epidemiological data we verified if the strains from the same cluster were truly involved in transmission events. We found that PFGE has lower capacity to correctly identify strains involved in clusters, while both WGS approaches showed better resolution (Table 2). In the absence of fixed thresholds, the best approach is probably the use of both analysis methods, supported by the epidemiological investigation.

We also compared the applicability of cgMLST genes and coreSNP for phylogenetic reconstruction. We analysed two datasets, the first including the 80 strains isolated from the OSR (OSR dataset), and a larger dataset including the same 80 strains plus additional 406 from public databases (Global dataset). While the cgMLST and coreSNP phylogenetic trees obtained for the OSR dataset were comparable (Fig. 3), the two trees for the Global dataset present important differences (Fig. 4). cgMLST wrongly placed CG258 strains, in particular ‘ST258_Clade1’ and ‘ST512/ST258_Clade2’ strains (Fig. 4). The ST258 emerged after a ~1 Mb recombination [52]. Then, a second omologous recombination of a ~215 kb genomic region, including the capsule polysaccharide synthesis (*cps*) locus, divided the ST258 in two sub-clades: ST258_Clade1 and ST258_Clade2 (which include also the ST512) [53]. The *cps* locus is a major source of variability in *K. pneumoniae* and the *wzi* gene is used to differentiate capsular types. For instance, *bla*_KPC-3_ and *wzi154* variants are strongly associated with ST258_Clade2, while *bla*_KPC-2_ and *wzi29* variants are associated with ST258_Clade1 [51, 54]. The correct attribution of a strain to ST258 Clade1 and Clade2 could be of pivotal epidemiological importance.

Considering that both Global trees are generated using the same evolutionary model (GTR), the misplacement of CG258 strains in the cgMLST Global tree could be due to the low number of cgMLST genes localized inside the first half of the ~1 Mb recombined region described by [53], which likely contains genetic information important to correctly reconstruct the phylogenetic tree of the CG258 (see Fig. S4).

In conclusion, we showed that, in our setting, both cgMLST and coreSNP analyses are more discriminatory than PFGE. Both are suitable for epidemiological investigations nonetheless we suggest to perform clustering analysis considering a range of thresholds or combining both the methodologies. The most important difference between coreSNP and cgMLST is that coreSNP-based approach shows a higher capacity to perform a proper CG258 clade discrimination compared to cgMLST in phylogenetic reconstructions.

## Data Bibliography

1. Navon-Venezia S, Kondratyeva K, Carattoli A. Klebsiella pneumoniae: a major worldwide source and shuttle for antibiotic resistance. *FEMS Microbiol Rev*. 2017 May 1; 41(3):252–275. doi: 10.1093/femsre/fux013

2. Wyres KL, Wick RR, Judd LM, Froumine R, Tokolyi A, Gorrie CL, Lam MMC, Duchêne S, Jenney A, Holt KE. Distinct evolutionary dynamics of horizontal gene transfer in drug resistant and virulent clones of Klebsiella pneumoniae. *PLoS Genet*. 2019 Apr 15; 15(4):e1008114. doi: 10.1371/journal.pgen.1008114.

3. Bialek-Davenet, S. *et al*. (2014) Genomic definition of hypervirulent and multidrug-resistant Klebsiella pneumoniae clonal groups. *Emerg Infect Dis*. 2014 Nov; 20(11):1812–20. doi: 10.3201/eid2011.140206.

4. Dekker JP, Frank KM. Next-generation epidemiology: using real-time core genome multilocus sequence typing to support infection control policy. *J Clin Microbiol*. 2016 Dec;54(12):2850–2853 doi: 10.1128/JCM.01714-16.

5. Onori, R., Gaiarsa, S., Comandatore, F., Pongolini, S., Brisse, S., Colombo, A., *et al*. Tracking nosocomial Klebsiella pneumoniae infections and outbreaks by whole-genome analysis: small-scale italian scenario within a single hospital. *J Clin Microbiol.* 2015 Sep;53(9):2861–8. doi: 10.1128/JCM.00545-15.

6. David S *et al.* Epidemic of carbapenem-resistant Klebsiella pneumoniae in Europe is driven by nosocomial spread. *Nat Microbiol.* 2019 Nov; 4(11):1919–1929. doi: 10.1038/s41564-019-0492-8.

## Supplementary Data

Supplementary material 1
