# Supplementary material for: Comparison of core-genome MLST, coreSNP and PFGE methods for Klebsiella pneumoniae cluster analysis
Source: Microb Genom. 2020 Mar 9;6(4):e000347. doi: 10.1099/mgen.0.000347 (PMC7276701; doi:10.1099/mgen.0.000347)
Supplement: Supplementary material 1 [file mgen-6-347-s001.pdf]

Table.S1 Statistics about genome assemblies and the number of undetermined cgMLST genes.

Strain	Source	Num. Contigs >5000nt	Total length contigs >5000nt	cgMLST undetermined genes
KP10	This study	63	5469150	3
KP118	This study	93	5900135	7
KP119	This study	53	5455189	4
KP12	This study	49	5538580	4
KP122	This study	54	5413779	6
KP123	This study	70	5519583	4
KP124	This study	51	5543909	8
KP125	This study	57	5358443	26
KP126	This study	61	5451288	3
KP127	This study	84	5594169	5
KP2	This study	69	5390131	3
KP232	This study	75	5602725	5
KP234	This study	70	5436720	6
KP236	This study	57	5318367	4
KP238	This study	63	5418368	3
KP239	This study	82	5467899	6
KP240	This study	102	5447076	3
KP242	This study	106	5359126	5
KP243	This study	60	5424968	5
KP245	This study	76	5446763	9
KP246	This study	82	5414024	30
KP248	This study	142	5480748	3
KP249	This study	120	5396241	6
KP251	This study	53	5518155	4
KP252	This study	81	5623534	6
KP255	This study	69	5560861	7
KP256	This study	93	5456742	6
KP257	This study	100	5542737	6
KP258	This study	78	5481445	3
KP260	This study	91	5610039	4
KP261	This study	72	5460086	8
KP265	This study	56	5395249	3
KP355	This study	55	5432317	4
KP356	This study	41	5253118	26
KP359	This study	55	5604354	3
KP360	This study	85	5447294	5
KP361	This study	116	5497119	4
KP363	This study	95	5568614	6
KP364	This study	122	5455006	5
KP365	This study	74	5564109	6
KP366	This study	91	5487491	3
KP367	This study	83	5446977	4
KP368	This study	120	5360681	10
KP4	This study	65	5476715	3
KP468	This study	84	5577561	4
KP469	This study	103	5870349	7
KP471	This study	67	5460793	6
KP472	This study	114	5403837	6
KP473	This study	86	5833616	6

KP475	This study	98	5478890	5
KP476	This study	61	5520699	3
KP477	This study	80	5241849	3
KP478	This study	73	5505423	4
KP479	This study	64	5308288	4
KP480	This study	66	5479009	3
KP481	This study	91	5414779	4
KP485	This study	98	5820952	3
KP486	This study	111	5460722	6
KP488	This study	87	5615474	5
KP489	This study	71	5423187	5
KP491	This study	83	5449124	3
KP492	This study	93	5591087	5
KP493	This study	89	5696080	3
KP496	This study	72	5498351	5
KP498	This study	94	5411261	5
KP499	This study	71	5593266	6
KP501	This study	86	5633238	5
KP502	This study	144	5332524	6
KP596	This study	67	5440892	6
KP597	This study	94	5416549	7
KP598	This study	98	5528905	4
KP599	This study	72	5640546	3
KP601	This study	95	5529444	4
KP603	This study	97	5540826	6
KP604	This study	98	5476989	4
KP605	This study	90	5452722	4
KP606	This study	100	5499991	19
KP613	This study	87	5408609	6
KP715	This study	90	5446230	4
KP9	This study	83	5580108	4
1037890.3	PATRIC database	62	5452783	6
1087440.3	PATRIC database	69	5630407	4
1087443.3	PATRIC database	90	5605308	5
1087444.3	PATRIC database	89	5608212	8
1087445.3	PATRIC database	79	5608995	5
1094167.3	PATRIC database	75	5602612	7
1094168.3	PATRIC database	80	5578480	8
1094169.3	PATRIC database	63	5631201	6
1094170.3	PATRIC database	76	5566912	23
1094171.3	PATRIC database	81	5563870	11
1128946.3	PATRIC database	75	5591236	14
1128948.3	PATRIC database	83	5603653	28

1128950.3	PATRIC database	71	5567132	9
1128951.3	PATRIC database	68	5613295	6
1128952.3	PATRIC database	76	5645422	14
1128953.3	PATRIC database	98	5582111	54
1128954.3	PATRIC database	73	5595842	20
1168062.3	PATRIC database	87	5623936	6
1168063.3	PATRIC database	84	5645820	4
1168064.3	PATRIC database	174	5513024	28
1177153.3	PATRIC database	86	5310294	6
1212766.3	PATRIC database	138	5385231	6
1226115.3	PATRIC database	31	5351459	4
1236102.6	PATRIC database	240	5407167	74
1267897.3	PATRIC database	167	5228674	5
1290996.3	PATRIC database	56	5623682	4
1294138.3	PATRIC database	63	5612262	4
1294139.3	PATRIC database	60	5613223	5
1294140.3	PATRIC database	65	5547712	7
1294141.3	PATRIC database	64	5566640	5
1381121.4	PATRIC database	43	5625953	7
1384549.3	PATRIC database	96	5751958	33
1384550.3	PATRIC database	90	5717009	6
1406314.3	PATRIC database	5	5812304	2
1413252.3	PATRIC database	91	5691953	5
1413253.3	PATRIC database	88	5728315	32
1420013.3	PATRIC database	4	5417216	15
1455605.3	PATRIC database	86	5543811	46
573.1345	PATRIC database	88	5368175	11
573.1347	PATRIC database	65	5497522	5
573.1351	PATRIC database	84	5408648	6
573.1352	PATRIC database	3	5510332	5
573.1358	PATRIC database	5	5990738	4

573.1359	PATRIC database	3	5556732	2
573.1361	PATRIC database	1	5118878	4
573.1366	PATRIC database	36	5520096	3
573.15221	PATRIC database	58	5553420	6
573.15228	PATRIC database	62	5545167	6
573.15432	PATRIC database	37	5298148	1
573.15433	PATRIC database	47	5417039	3
573.15435	PATRIC database	78	5783342	2
573.15438	PATRIC database	82	5783483	2
573.15439	PATRIC database	81	5784528	2
573.15440	PATRIC database	81	5776616	2
573.15442	PATRIC database	64	5367178	4
573.15445	PATRIC database	46	5339989	4
573.15447	PATRIC database	47	5499575	3
573.15448	PATRIC database	80	5800050	2
573.15452	PATRIC database	44	5304295	6
573.15455	PATRIC database	43	5338240	3
573.15459	PATRIC database	51	5396165	3
573.15461	PATRIC database	82	5785518	2
573.15462	PATRIC database	46	5428187	3
573.15463	PATRIC database	47	5383532	1
573.15466	PATRIC database	84	5834920	2
573.15467	PATRIC database	80	5788314	2
573.15468	PATRIC database	65	5434230	5
573.15470	PATRIC database	42	5420495	1
573.15471	PATRIC database	46	5328468	5
573.15472	PATRIC database	55	5562669	2
573.15474	PATRIC database	53	5453670	3
573.15477	PATRIC database	32	5237913	3
573.15478	PATRIC database	79	5794514	2
573.15482	PATRIC database	49	5419623	2

573.15483	PATRIC database	36	5336924	3
573.15485	PATRIC database	66	5434907	4
573.15487	PATRIC database	54	5530888	2
573.15489	PATRIC database	79	5774960	2
573.15490	PATRIC database	78	5783909	2
573.15496	PATRIC database	80	5780338	2
573.15500	PATRIC database	40	5374929	3
573.15502	PATRIC database	81	5835446	2
573.15506	PATRIC database	36	5408619	4
573.15509	PATRIC database	66	5550490	4
573.15510	PATRIC database	40	5412710	3
573.15512	PATRIC database	25	5097183	4
573.15513	PATRIC database	32	5270972	4
573.15518	PATRIC database	47	5506373	1
573.15521	PATRIC database	37	5478071	2
573.15522	PATRIC database	24	5146478	3
573.15525	PATRIC database	26	5112960	1
573.15536	PATRIC database	28	5239858	1
573.15540	PATRIC database	32	5166370	4
573.15546	PATRIC database	40	5406836	2
573.15549	PATRIC database	49	5338293	2
573.15560	PATRIC database	29	5113594	3
573.15574	PATRIC database	30	5184401	2
573.15577	PATRIC database	31	5147994	4
573.15579	PATRIC database	35	5361824	6
573.15580	PATRIC database	38	5397620	2
573.15583	PATRIC database	26	5238382	1
573.15585	PATRIC database	37	5108122	3
573.15588	PATRIC database	28	5360855	0
573.15591	PATRIC database	29	5360999	0
573.15593	PATRIC database	39	5386109	2

573.15595	PATRIC database	47	5387190	2
573.15596	PATRIC database	38	5252579	2
573.15598	PATRIC database	27	5041433	3
573.15599	PATRIC database	54	5387960	2
573.15603	PATRIC database	84	5539993	5
573.15605	PATRIC database	25	5393979	2
573.15607	PATRIC database	50	5566147	3
573.15610	PATRIC database	39	5362612	2
573.15613	PATRIC database	40	5375125	2
573.15614	PATRIC database	41	5388024	2
573.15615	PATRIC database	36	5120263	0
573.15618	PATRIC database	39	5399852	2
573.15629	PATRIC database	76	5535709	5
573.15635	PATRIC database	60	5496066	3
573.15647	PATRIC database	54	5527809	3
573.15654	PATRIC database	53	5531735	3
573.15656	PATRIC database	53	5527623	3
573.15657	PATRIC database	50	5531383	4
573.15658	PATRIC database	51	5416095	3
573.15659	PATRIC database	53	5417293	3
573.15661	PATRIC database	51	5386337	3
573.15662	PATRIC database	53	5419610	3
573.15664	PATRIC database	82	5275249	5
573.15665	PATRIC database	54	5415201	3
573.15666	PATRIC database	50	5383258	3
573.15667	PATRIC database	53	5421938	3
573.15668	PATRIC database	50	5425306	3
573.15669	PATRIC database	50	5410838	3
573.15670	PATRIC database	53	5420393	3
573.15671	PATRIC database	52	5422691	3
573.15672	PATRIC database	54	5415939	3

573.15673	PATRIC database	54	5415760	3
573.15674	PATRIC database	54	5420324	3
573.15675	PATRIC database	52	5420243	3
573.15711	PATRIC database	30	5223979	4
573.15713	PATRIC database	71	5502203	4
573.15714	PATRIC database	56	5420508	3
573.15727	PATRIC database	66	5490649	3
573.15729	PATRIC database	67	5491581	3
573.16446	PATRIC database	97	5667481	8
573.1719	PATRIC database	76	5704748	8
573.1720	PATRIC database	63	5531676	8
573.1721	PATRIC database	65	5377098	8
573.1722	PATRIC database	59	5391850	7
573.1723	PATRIC database	86	5595167	11
573.1724	PATRIC database	75	5567939	6
573.1725	PATRIC database	84	5590266	9
573.1726	PATRIC database	81	5621863	7
573.1727	PATRIC database	73	5449065	5
573.1728	PATRIC database	65	5623891	4
573.1729	PATRIC database	88	5610130	5
573.1730	PATRIC database	70	5469153	6
573.1731	PATRIC database	71	5631637	6
573.1732	PATRIC database	80	5612097	5
573.1733	PATRIC database	70	5692493	5
573.1734	PATRIC database	62	5603359	11
573.17823	PATRIC database	61	5407567	16
573.17824	PATRIC database	75	5395625	4
573.17825	PATRIC database	78	5417074	4
573.17826	PATRIC database	74	5418170	6
573.18541	PATRIC database	4	6107937	8
573.1925	PATRIC database	35	5352255	4

573.1926	PATRIC database	60	5237620	6
573.1927	PATRIC database	44	5345828	2
573.1928	PATRIC database	41	5348567	2
573.1929	PATRIC database	38	5355732	2
573.1930	PATRIC database	35	5357074	2
573.1931	PATRIC database	42	5345931	2
573.1932	PATRIC database	45	5184369	11
573.1933	PATRIC database	37	5353995	3
573.1934	PATRIC database	57	5401301	6
573.1935	PATRIC database	42	5346939	2
573.1936	PATRIC database	52	5344017	2
573.20074	PATRIC database	49	5402233	5
573.2030	PATRIC database	66	5492650	31
573.2031	PATRIC database	85	5491106	31
573.2032	PATRIC database	80	5493904	31
573.2033	PATRIC database	63	5501396	32
573.2034	PATRIC database	75	5501127	31
573.2035	PATRIC database	67	5493135	31
573.2036	PATRIC database	70	5506426	31
573.2300	PATRIC database	59	5438904	6
573.2301	PATRIC database	82	5366132	12
573.2302	PATRIC database	56	5446235	4
573.2303	PATRIC database	61	5440779	8
573.2304	PATRIC database	57	5436427	5
573.2305	PATRIC database	72	5412841	7
573.2306	PATRIC database	56	5298223	2
573.2308	PATRIC database	71	5359371	7
573.2309	PATRIC database	43	5434504	5
573.2310	PATRIC database	54	5447549	6
573.2311	PATRIC database	70	5436225	6
573.2312	PATRIC database	55	5452933	6
573.2313	PATRIC database	54	5441459	5

573.2314	PATRIC database	62	5448293	2
573.2315	PATRIC database	59	5446047	2
573.2316	PATRIC database	59	5443256	2
573.2317	PATRIC database	76	5436984	2
573.4042	PATRIC database	36	5299007	11
573.4044	PATRIC database	52	5568015	11
573.4046	PATRIC database	33	5268170	6
573.4051	PATRIC database	20	5180757	7
573.4058	PATRIC database	43	5549772	7
573.4061	PATRIC database	41	5534724	5
573.4064	PATRIC database	34	5440999	9
573.4071	PATRIC database	23	5321511	9
573.4072	PATRIC database	45	5347609	3
573.4077	PATRIC database	36	5591900	3
573.4089	PATRIC database	25	5442919	6
573.4093	PATRIC database	34	5381493	7
573.4100	PATRIC database	37	5524399	13
573.4101	PATRIC database	36	5376919	4
573.4102	PATRIC database	35	5303133	7
573.4103	PATRIC database	45	5439626	6
573.4104	PATRIC database	24	5500358	7
573.4110	PATRIC database	33	5543310	7
573.4111	PATRIC database	36	5458582	3
573.4114	PATRIC database	26	5464180	6
573.4116	PATRIC database	21	5470057	15
573.4122	PATRIC database	39	5583227	8
573.4124	PATRIC database	33	5487639	34
573.4126	PATRIC database	15	5374571	5
573.4127	PATRIC database	44	5360968	32
573.4129	PATRIC database	36	5376663	7
573.4130	PATRIC database	42	5526691	10

573.4132	PATRIC database	31	5382028	5
573.4133	PATRIC database	26	5357087	4
573.4134	PATRIC database	35	5304033	10
573.4135	PATRIC database	31	5441414	5
573.4136	PATRIC database	37	5565353	8
573.4137	PATRIC database	30	5413615	8
573.4138	PATRIC database	36	5297465	11
573.4141	PATRIC database	23	5475559	7
573.4143	PATRIC database	37	5434392	4
573.4144	PATRIC database	45	5287757	11
573.4146	PATRIC database	34	5363684	6
573.4148	PATRIC database	37	5368278	5
573.4149	PATRIC database	26	5511397	9
573.4157	PATRIC database	33	5521251	7
573.4158	PATRIC database	30	5446359	7
573.4159	PATRIC database	34	5329607	6
573.4162	PATRIC database	30	5271359	20
573.4164	PATRIC database	51	5509888	5
573.4165	PATRIC database	49	5580245	6
573.4166	PATRIC database	39	5505287	35
573.4167	PATRIC database	28	5458334	7
573.4170	PATRIC database	24	5346901	8
573.4171	PATRIC database	30	5523069	8
573.4173	PATRIC database	48	5486495	4
573.4177	PATRIC database	52	5469411	24
573.4179	PATRIC database	31	5214480	6
573.4183	PATRIC database	31	5293893	3
573.4184	PATRIC database	34	5528142	7
573.4185	PATRIC database	32	5465240	8
573.4193	PATRIC database	44	5480997	28
573.4196	PATRIC database	46	5325790	18

573.4197	PATRIC database	36	5336415	17
573.4198	PATRIC database	44	5536946	29
573.4201	PATRIC database	30	5579052	8
573.4202	PATRIC database	39	5446266	8
573.4203	PATRIC database	45	5596063	6
573.4209	PATRIC database	37	5548066	34
573.4211	PATRIC database	31	5430186	5
573.4214	PATRIC database	26	5393074	8
573.4216	PATRIC database	38	5491410	4
573.4218	PATRIC database	38	5402065	6
573.4220	PATRIC database	29	5424918	12
573.4222	PATRIC database	29	5328855	5
573.4223	PATRIC database	12	4988911	6
573.4224	PATRIC database	31	5549146	5
573.4225	PATRIC database	27	5521488	6
573.4227	PATRIC database	20	5427877	20
573.4228	PATRIC database	32	5263935	31
573.5661	PATRIC database	17	5293004	0
573.5665	PATRIC database	28	5544898	2
573.5672	PATRIC database	34	5482155	5
573.5677	PATRIC database	31	5606011	4
573.5679	PATRIC database	38	5675185	2
573.5683	PATRIC database	24	5371013	2
573.5687	PATRIC database	21	5597358	6
573.5690	PATRIC database	25	5644540	3
573.5691	PATRIC database	28	5392181	3
573.5694	PATRIC database	19	5241603	4
573.5695	PATRIC database	22	5634451	3
573.5700	PATRIC database	40	5489594	4
573.5701	PATRIC database	17	5486234	6
573.5702	PATRIC database	22	5331779	2

573.5705	PATRIC database	19	5542035	3
573.5706	PATRIC database	22	5375613	1
573.5707	PATRIC database	15	5608600	3
573.5710	PATRIC database	28	5369956	3
573.5711	PATRIC database	18	5253529	2
573.5717	PATRIC database	28	5474982	3
573.5722	PATRIC database	30	5463867	3
573.5724	PATRIC database	16	5293492	1
573.5760	PATRIC database	81	5631051	5
573.5794	PATRIC database	31	5753547	3
573.5795	PATRIC database	27	5641183	3
573.5798	PATRIC database	40	5509930	4
573.5799	PATRIC database	45	5599954	4
573.5803	PATRIC database	25	5397227	3
573.5806	PATRIC database	21	5383177	3
573.5807	PATRIC database	16	5474921	6
573.5810	PATRIC database	16	5407801	4
573.5817	PATRIC database	14	5483614	6
573.5828	PATRIC database	48	5473062	3
573.7038	PATRIC database	31	5464799	8
573.7039	PATRIC database	39	5484571	29
573.7040	PATRIC database	49	5560469	21
573.7041	PATRIC database	33	5357476	8
573.7043	PATRIC database	54	5549485	8
573.7045	PATRIC database	23	5435010	5
573.7047	PATRIC database	25	5336598	10
573.7048	PATRIC database	42	5491013	5
573.7049	PATRIC database	43	5462419	8
573.7050	PATRIC database	43	5561082	7
573.7054	PATRIC database	27	5538087	11
573.7058	PATRIC database	29	5427156	5

573.7059	PATRIC database	48	5566292	12
573.7062	PATRIC database	25	5382366	11
573.7063	PATRIC database	34	5419436	7
573.7065	PATRIC database	40	5250222	41
573.7066	PATRIC database	23	5553098	11
573.7067	PATRIC database	39	5550044	8
573.7068	PATRIC database	36	5555615	7
573.7069	PATRIC database	37	5438087	9
573.7070	PATRIC database	21	5259624	8
573.7074	PATRIC database	45	5625542	6
573.7076	PATRIC database	46	5451743	8
573.7078	PATRIC database	39	5458764	7
573.7079	PATRIC database	38	5438667	9
573.7080	PATRIC database	27	5396056	8
573.7081	PATRIC database	34	5401211	5
573.7082	PATRIC database	55	5501534	9
573.7083	PATRIC database	26	5341318	7
573.7084	PATRIC database	28	5422077	11
573.7085	PATRIC database	32	5428539	8
573.7087	PATRIC database	33	5517732	12
573.7089	PATRIC database	37	5299321	15
573.7091	PATRIC database	29	5437370	10
573.7092	PATRIC database	48	5462148	13
573.7094	PATRIC database	48	5434574	18
573.7217	PATRIC database	50	5502999	5
573.7218	PATRIC database	61	5531411	5
573.7219	PATRIC database	47	5487957	16
573.7221	PATRIC database	50	5513584	5
573.7223	PATRIC database	46	5517074	5
573.7384	PATRIC database	42	5466561	3
573.9731	PATRIC database	40	5628479	6

573.9734	PATRIC database	36	5480750	3
573.9776	PATRIC database	75	5459816	9
72407.105	PATRIC database	151	5720077	24
72407.122	PATRIC database	1	5439720	9
72407.123	PATRIC database	1	5355459	10
72407.148	PATRIC database	68	5620067	4
72407.152	PATRIC database	75	5439887	4
72407.153	PATRIC database	73	5508104	4
72407.165	PATRIC database	69	5512200	30
72407.167	PATRIC database	80	5503453	4
72407.168	PATRIC database	72	5530432	4
72407.170	PATRIC database	76	5498728	5
72407.171	PATRIC database	78	5482457	4
72407.175	PATRIC database	73	5425019	4
72407.179	PATRIC database	68	5474670	4
72407.186	PATRIC database	72	5469362	5
72407.189	PATRIC database	62	5342341	9
72407.191	PATRIC database	84	5496282	5
72407.193	PATRIC database	64	5408074	4
72407.194	PATRIC database	74	5461419	4
72407.197	PATRIC database	68	5425097	6
72407.199	PATRIC database	75	5381886	8
72407.203	PATRIC database	78	5476957	5
72407.204	PATRIC database	79	5344680	5
72407.205	PATRIC database	81	5473222	5
72407.211	PATRIC database	82	5460774	5
72407.219	PATRIC database	70	5446504	5
72407.221	PATRIC database	79	5630573	5
72407.224	PATRIC database	78	5461507	5
72407.227	PATRIC database	70	5373160	5
72407.237	PATRIC database	64	5401980	4
72407.238	PATRIC database	160	5300183	30

72407.240	PATRIC database	75	5526293	4
72407.242	PATRIC database	67	5363209	6
72407.247	PATRIC database	72	5546624	5
72407.248	PATRIC database	72	5578003	4
72407.249	PATRIC database	63	5435984	5
72407.256	PATRIC database	81	5506787	5
72407.257	PATRIC database	68	5336317	30
72407.258	PATRIC database	82	5513265	6
72407.259	PATRIC database	80	5469898	5
72407.261	PATRIC database	87	5478183	5
72407.263	PATRIC database	81	5429867	11
72407.265	PATRIC database	73	5432317	5
72407.268	PATRIC database	76	5450473	6
72407.269	PATRIC database	79	5501352	4
72407.270	PATRIC database	77	5497383	5
72407.271	PATRIC database	74	5393867	4
72407.464	PATRIC database	5	5804404	7
72407.76	PATRIC database	4	5858308	4
72407.78	PATRIC database	4	5484149	4
72407.80	PATRIC database	53	5632347	4

Table S2. Accession numbers of 80 *K. pneumoniae* isolates

Strain name	MLST profile	Bioproject_Accession	BioSample_Accession	SRA Accession
**KP468**	**ST512**	PRJNA564099	SAMN12701027	SRX6820061
**KP469**	**ST395**	PRJNA564099	SAMN12701027	SRX6820062
**KP12**	**ST307**	PRJNA564099	SAMN12701027	SRX6820128
**KP366**	**ST11**	PRJNA564099	SAMN12701027	SRX6820117
**KP4**	**ST307**	PRJNA564099	SAMN12701027	SRX6820139
**KP471**	**ST307**	PRJNA564099	SAMN12701027	SRX6820074
**KP472**	**ST307**	PRJNA564099	SAMN12701027	SRX6820085
**KP473**	**ST101**	PRJNA564099	SAMN12701027	SRX6820096
**KP10**	**ST307**	PRJNA564099	SAMN12701027	SRX6820107
**KP475**	**ST307**	PRJNA564099	SAMN12701027	SRX6820108
**KP2**	**ST307**	PRJNA564099	SAMN12701027	SRX6820063
**KP9**	**ST512**	PRJNA564099	SAMN12701027	SRX6820064
**KP242**	**ST512**	PRJNA564099	SAMN12701027	SRX6820109
**KP243**	**ST307**	PRJNA564099	SAMN12701027	SRX6820110
**KP476**	**ST307**	PRJNA564099	SAMN12701027	SRX6820111
**KP119**	**ST307**	PRJNA564099	SAMN12701027	SRX6820112
**KP118**	**ST395**	PRJNA564099	SAMN12701027	SRX6820113
**KP478**	**ST512**	PRJNA564099	SAMN12701027	SRX6820114
**KP123**	**ST512**	PRJNA564099	SAMN12701027	SRX6820115
**KP122**	**ST307**	PRJNA564099	SAMN12701027	SRX6820116
**KP245**	**ST307**	PRJNA564099	SAMN12701027	SRX6820118
**KP258**	**ST307**	PRJNA564099	SAMN12701027	SRX6820119
**KP236**	**ST307**	PRJNA564099	SAMN12701027	SRX6820120
**KP480**	**ST307**	PRJNA564099	SAMN12701027	SRX6820121
**KP124**	**ST101**	PRJNA564099	SAMN12701027	SRX6820122
**KP125**	**ST307**	PRJNA564099	SAMN12701027	SRX6820123
**KP481**	**ST307**	PRJNA564099	SAMN12701027	SRX6820124
**KP127**	**512**	PRJNA564099	SAMN12701027	SRX6820125
**KP234**	**ST307**	PRJNA564099	SAMN12701027	SRX6820126
**KP126**	**ST307**	PRJNA564099	SAMN12701027	SRX6820127
**KP248**	**ST423**	PRJNA564099	SAMN12701027	SRX6820129
**KP246**	**ST258**	PRJNA564099	SAMN12701027	SRX6820130
**KP232**	**ST258**	PRJNA564099	SAMN12701027	SRX6820131
**KP486**	**ST307**	PRJNA564099	SAMN12701027	SRX6820132
**KP239**	**ST512**	PRJNA564099	SAMN12701027	SRX6820133

**KP238**	**ST307**	PRJNA564099	SAMN12701027	SRX6820134
**KP240**	**ST307**	PRJNA564099	SAMN12701027	SRX6820135
**KP488**	**ST258**	PRJNA564099	SAMN12701027	SRX6820136
**KP249**	**ST512**	PRJNA564099	SAMN12701027	SRX6820137
**KP251**	**ST307**	PRJNA564099	SAMN12701027	SRX6820138
**KP489**	**ST512**	PRJNA564099	SAMN12701027	SRX6820140
**KP252**	**ST258**	PRJNA564099	SAMN12701027	SRX6820065
**KP265**	**ST307**	PRJNA564099	SAMN12701027	SRX6820066
**KP491**	**ST307**	PRJNA564099	SAMN12701027	SRX6820067
**KP261**	**ST258**	PRJNA564099	SAMN12701027	SRX6820068
**KP256**	**ST512**	PRJNA564099	SAMN12701027	SRX6820069
**KP257**	**ST512**	PRJNA564099	SAMN12701027	SRX6820070
**KP492**	**ST512**	PRJNA564099	SAMN12701027	SRX6820071
**KP255**	**ST101**	PRJNA564099	SAMN12701027	SRX6820072
**KP356**	**ST307**	PRJNA564099	SAMN12701027	SRX6820073
**KP260**	**ST512**	PRJNA564099	SAMN12701027	SRX6820075
**KP355**	**ST512**	PRJNA564099	SAMN12701027	SRX6820076
**KP359**	**ST149**	PRJNA564099	SAMN12701027	SRX6820077
**KP496**	**ST512**	PRJNA564099	SAMN12701027	SRX6820078
**KP363**	**ST512**	PRJNA564099	SAMN12701027	SRX6820079
**KP360**	**ST512**	PRJNA564099	SAMN12701027	SRX6820080
**KP364**	**ST512**	PRJNA564099	SAMN12701027	SRX6820081
**KP498**	**ST512**	PRJNA564099	SAMN12701027	SRX6820082
**KP365**	**ST101**	PRJNA564099	SAMN12701027	SRX6820083
**KP361**	**ST512**	PRJNA564099	SAMN12701027	SRX6820084
**KP499**	**ST512**	PRJNA564099	SAMN12701027	SRX6820086
**KP367**	**ST512**	PRJNA564099	SAMN12701027	SRX6820087
**KP613**	**ST307**	PRJNA564099	SAMN12701027	SRX6820088
**KP368**	**ST512**	PRJNA564099	SAMN12701027	SRX6820089
**KP501**	**ST512**	PRJNA564099	SAMN12701027	SRX6820090
**KP502**	**ST512**	PRJNA564099	SAMN12701027	SRX6820091
**KP596**	**ST307**	PRJNA564099	SAMN12701027	SRX6820092
**KP597**	**ST101**	PRJNA564099	SAMN12701027	SRX6820093
**KP599**	**ST307**	PRJNA564099	SAMN12701027	SRX6820094
**KP601**	**ST512**	PRJNA564099	SAMN12701027	SRX6820095
**KP598**	**ST512**	PRJNA564099	SAMN12701027	SRX6820097
**KP603**	**ST512**	PRJNA564099	SAMN12701027	SRX6820098
**KP604**	**ST512**	PRJNA564099	SAMN12701027	SRX6820099

**KP715**	**ST512**	PRJNA564099	SAMN12701027	SRX6820100
**KP605**	**ST512**	PRJNA564099	SAMN12701027	SRX6820101
**KP606**	**ST307**	PRJNA564099	SAMN12701027	SRX6820102
**KP477**	**ST37**	PRJNA564099	SAMN12701027	SRX6820103
**KP479**	**ST307**	PRJNA564099	SAMN12701027	SRX6820104
**KP485**	**ST15**	PRJNA564099	SAMN12701027	SRX6820105
**KP493**	**ST307**	PRJNA564099	SAMN12701027	SRX6820106

Figure S1. Rarefaction curve of the core cgMLST genes in the Global dataset computed with 100 bootstrap replicates

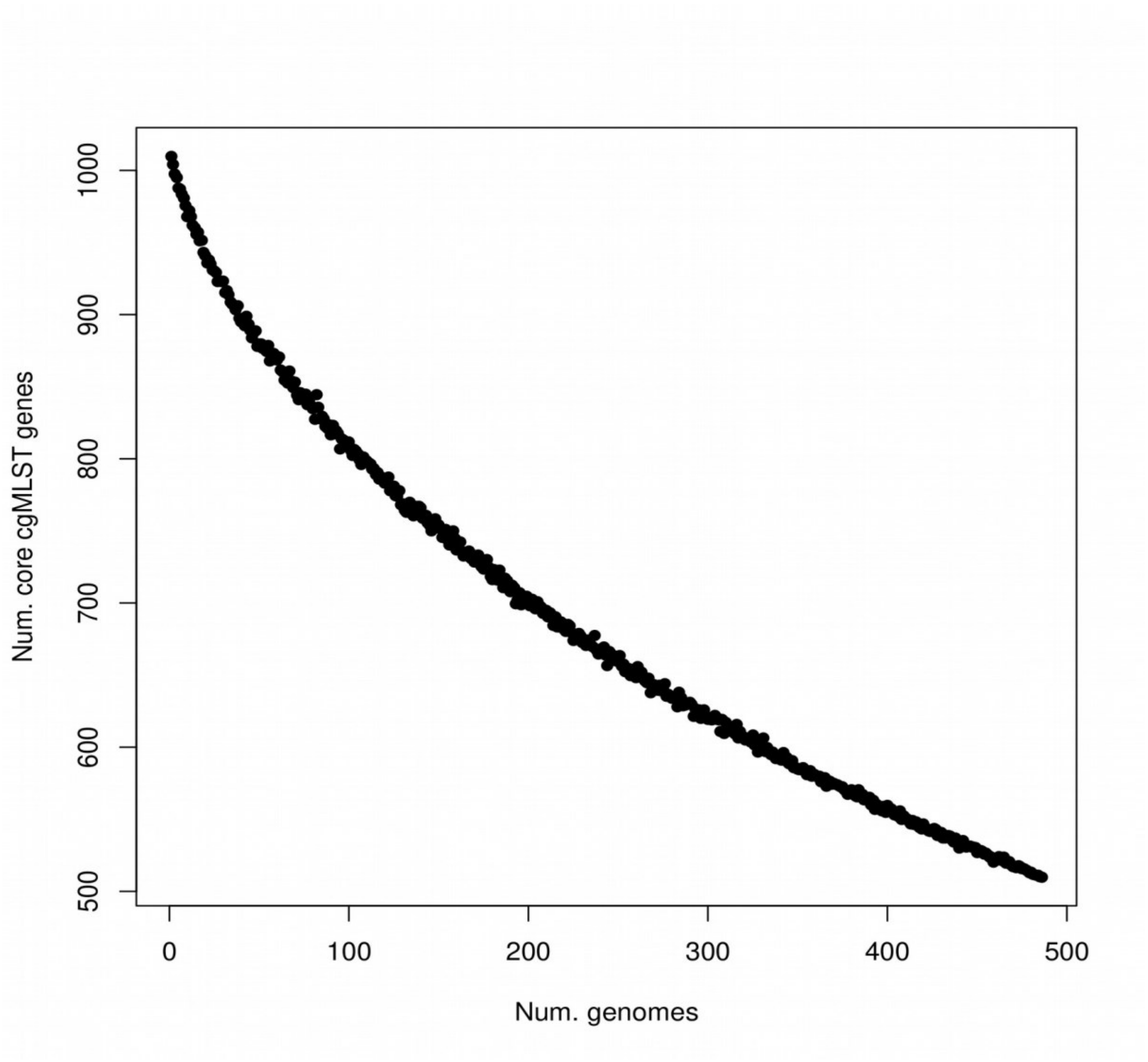

Figure S2. Rarefaction curve of the core cgMLST genes in the OSR dataset computed with 100 bootstrap replicates

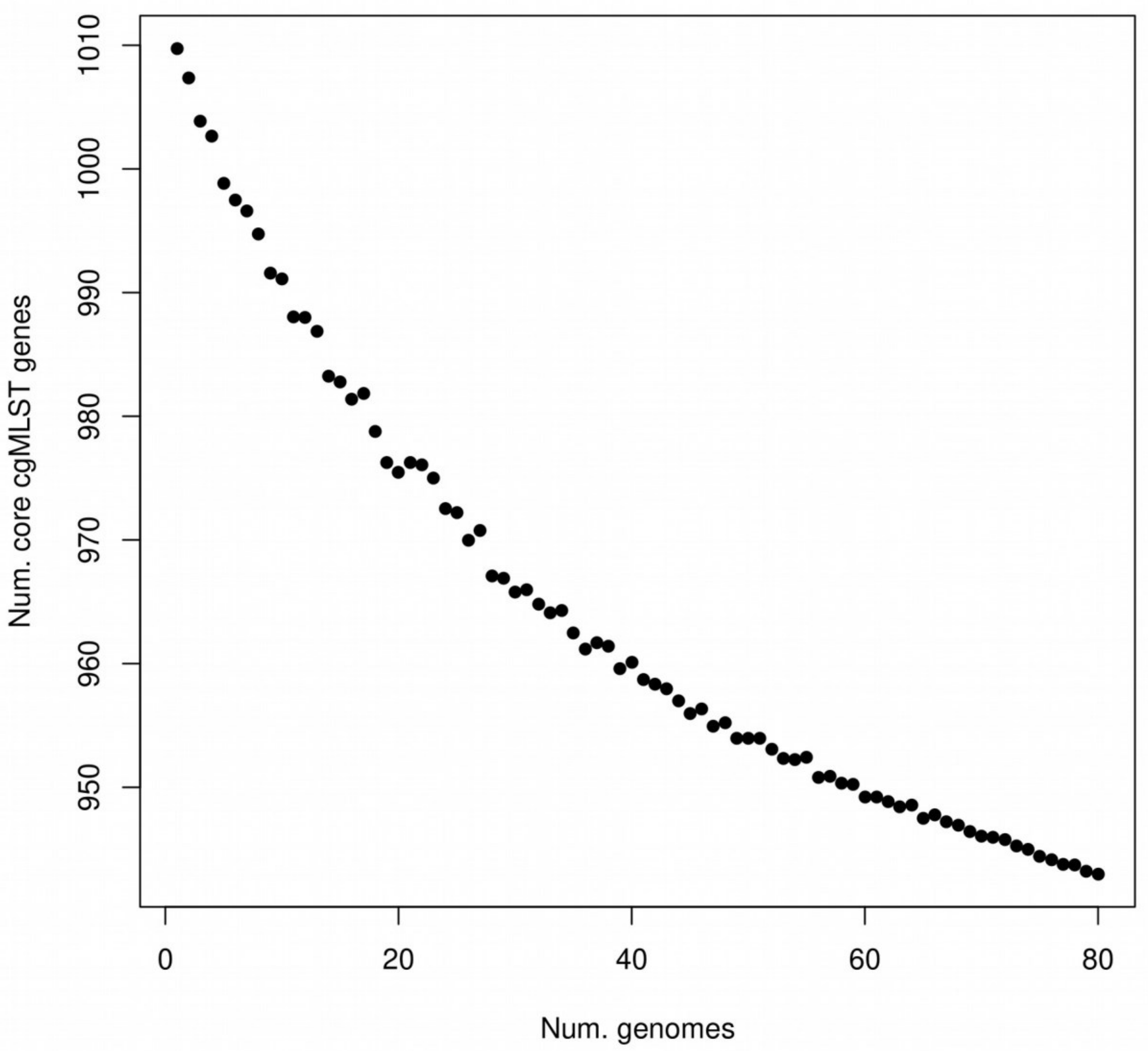

Figure S3. Scatter plot of the pairwise coreSNP distances and cgMSLT distances among the 486 Global dataset strains. The results of the Spearman regression analysis (R and p-value) are reported on the graph.

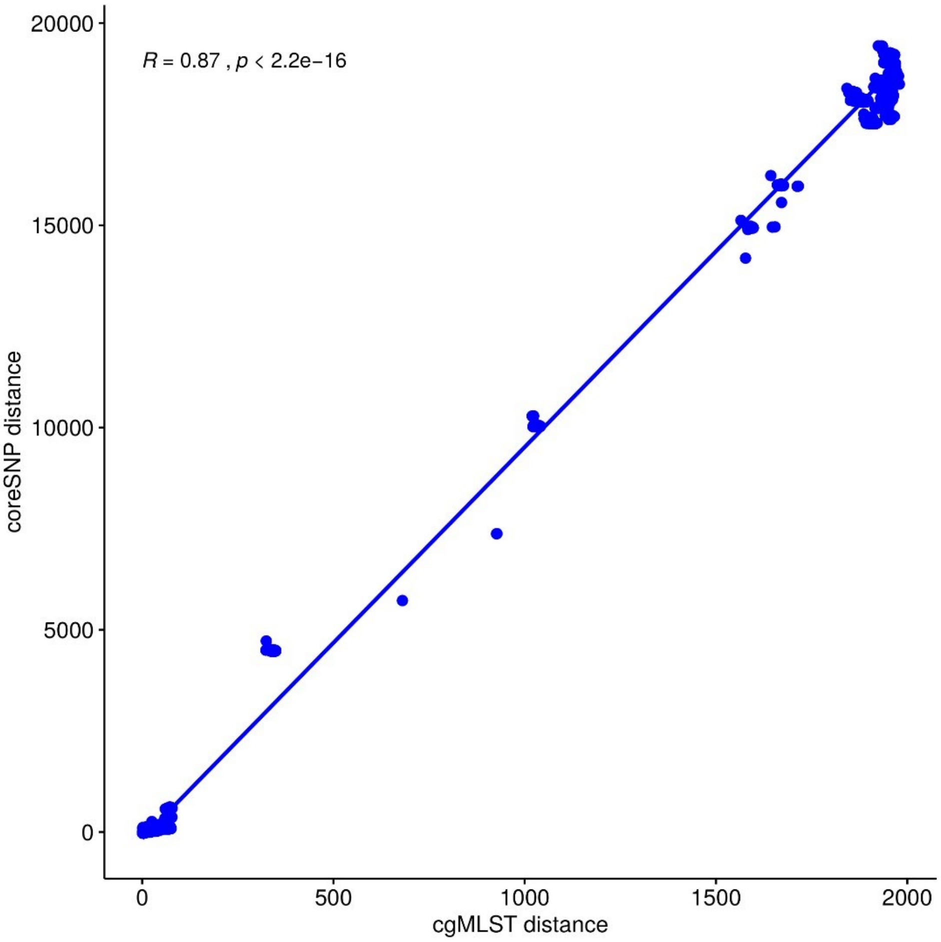

Figure S4. Distribution of coreSNP and cgMLST on the reference genome (NTUH-K2044) are reported as two histograms coloured in gray and red, respectively. At the bottom of the graph, the ~1Mb genomic position homologous to that involved by the recombination that led to the mergence of the ST258 lineage is reported in light red, while that involved by the ~215kb sized recombination evente that led to the emergence of the ST258_Clade2 lineage is reported in dark green.

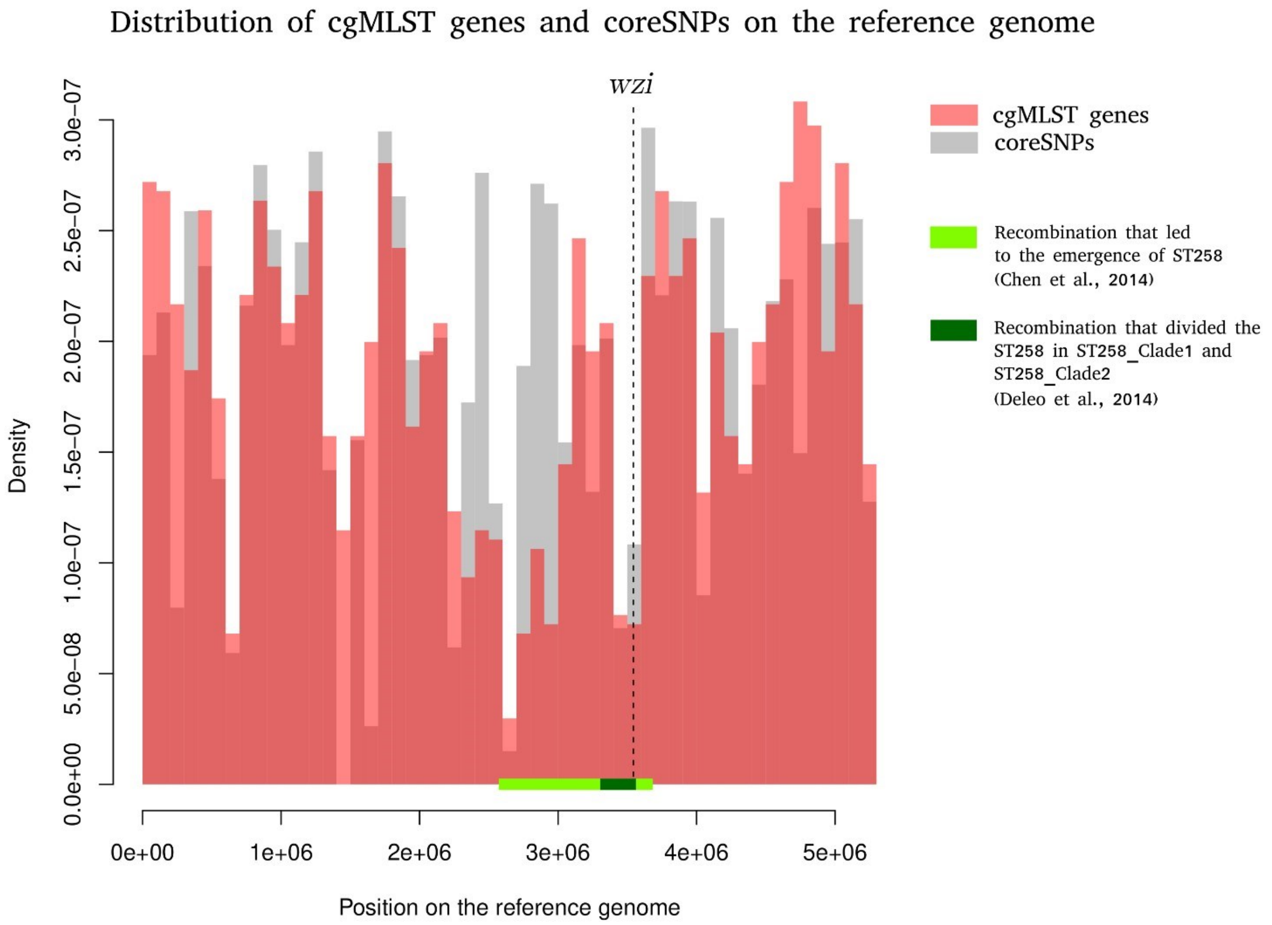

-*in-house* Perl script (https://drive.google.com/open?id=1OlSmcQmcm4-5hfSCu1bov3M8AXS96Xbt) used to concatenate the genes

```
use Bio::SeqIO;

$folder=shift;

@ls=`ls $folder/*`;


foreach $file(@ls)
{
chomp $file;

$in  = Bio::SeqIO->new(-file => $file , '-format' => 'Fasta');

while(my $seq = $in->next_seq)
{
	$hash{$seq->display_id} = $hash{$seq->display_id}.$seq->seq;
}
}

foreach $k(keys %hash)
{
print ">",$k,"\n",$hash{$k},"\n";
}
```
